# Identification of an inhibitory domain in GTPase-activating protein p190RhoGAP responsible for masking its functional GAP domain

**DOI:** 10.1016/j.jbc.2022.102792

**Published:** 2022-12-11

**Authors:** Capucine Héraud, Mathilde Pinault, Véronique Neaud, Frédéric Saltel, Valérie Lagrée, Violaine Moreau

**Affiliations:** Bordeaux Institute of Oncology, BRIC U1312, INSERM, Université de Bordeaux, Bordeaux, France

**Keywords:** GTPase-activating protein, conformational change, Rho (Rho GTPase), structure-function, cancer, mutant, actin, co-IP, coimmunoprecipitation, Cter, carboxy terminal, GAP, GTPase-activating protein, GBD, GTP-binding domain, Nter, amino terminal, PLS, protrusion localization sequence

## Abstract

The GTPase-activating protein (GAP) p190RhoGAP (p190A) is encoded by *ARHGAP35* which is found mutated in cancers. p190A is a negative regulator of the GTPase RhoA in cells and must be targeted to RhoA-dependent actin-based structures to fulfill its roles. We previously identified a functional region of p190A called the PLS (protrusion localization sequence) required for localization of p190A to lamellipodia but also for regulating the GAP activity of p190A. Additional effects of the PLS region on p190A localization and activity need further characterization. Here, we demonstrated that the PLS is required to target p190A to invadosomes. Cellular expression of a p190A construct devoid of the PLS (p190AΔPLS) favored RhoA inactivation in a stronger manner than WT p190A, suggesting that the PLS is an autoinhibitory domain of p190A GAP activity. To decipher this mechanism, we searched for PLS-interacting proteins using a two-hybrid screen. We found that the PLS can interact with p190A itself. Coimmunoprecipitation experiments demonstrated that the PLS interacts with a region in close proximity to the GAP domain. Furthermore, we demonstrated that this interaction is abolished if the PLS harbors cancer-associated mutations: the S866F point mutation and the Δ865–870 deletion. Our results are in favor of defining PLS as an inhibitory domain responsible for masking the p190A functional GAP domain. Thus, p190A could exist in cells under two forms: an inactive closed conformation with a masked GAP domain and an open conformation allowing p190A GAP function. Altogether, our data unveil a new mechanism of p190A regulation.

Small GTPases of the Rho family are molecular switches of key signaling pathways involved in cytoskeletal remodeling. A spatiotemporal regulation of Rho GTPases is required for achieving their key functions. For instance, efficient cell migration requires the sequential activation/inactivation of Rho GTPases, including RhoA, Rac1, and Cdc42, to generate cycles of membrane protrusion (*i.e.*, lamellipodia and filopodia) and adhesion site assembly (*i.e.*, stress fibers) (1, 2, 3). To this end, tight control of GTPase hydrolase activity is crucial, with one of the primary regulators of GDP/GTP cycling of Rho GTPases being GTPase-activating proteins (GAPs).

p190RhoGAP-A (also known as ARHGAP35 or GRLF1 and hereafter called p190A) is a GAP that acts on Rho GTPases. p190A plays important roles in actin regulatory pathways in numerous cell types and controls diverse cellular mechanisms, such as cell division, migration, and invasion, endothelial permeability, and cell death (4). The subcellular localization of p190A is highly regulated to fulfill these specific functions. Thus, p190A accumulates in actin-rich structures, including new adhesion sites (5), lamellipodia/ruffles (6, 7), invadosomes (8, 9), or cleavage furrows (10). Given the enzymatic activity of p190A enhances GTP hydrolysis, p190A is mainly involved in the attenuation of the biological function of RhoA GTPase. However, p190A may also function as a signal terminator for Rac1 and RhoC (4).

p190A is a large (≈1500 aa) multidomain protein with a C-terminal catalytic domain. In addition to this RhoGAP module, p190A contains three other structural domains. The N-terminal part of p190A harbors a GTP-binding domain (GBD), described recently as a class (ii) pseudo-GTPase domain, that is, a nucleotide-binding and catalytically inactive GTPase (11). There are also four motifs, the so-called FF motifs, in proximity to the GBD. These consist of 50 aa with two strictly conserved phenylalanine residues. Finally, the large domain between the FF and GAP domains contains two class (i) pseudo-GTPase domains (pG1 and pG2) that harbor neither nucleotide-binding activity nor catalytic activity (12). Besides these structural domains, several functional domains have been characterized in p190A. A binding site for p120RasGAP, a GAP of the Ras GTPases, was identified between the pG2 and the GAP domain. Two Src-phosphorylated tyrosine residues (Y1087 and Y1105) are involved in the regulation of this interaction (13, 14). Furthermore, we identified another functional region (residues 380–971), including two FF motifs, pG1 and pG2, implicated in the regulation of subcellular localization and function of p190A. This region, named protrusion localization sequence (PLS), is needed for the targeting of p190A to lamellipodia (6). Whether the PLS is also required for targeting p190A to other actin-rich subcellular structures, such as invadosomes, remains to be explored. Importantly, we also previously showed that the PLS regulates the GAP activity of p190A (6). A truncated protein devoid of the PLS (p190ΔPLS) exhibited an increased ability to constitutively bind active RhoA in comparison to the WT protein. How the PLS regulates p190A activity is unknown and is the focus of the present study.

p190A recently regained attention after being found mutated in cancers. Several large-scale studies mapping somatic variants across thousands of tumors identified *ARHGAP35*—the gene encoding p190A–as a new major cancer gene (15, 16). *ARHGAP35* appears mutated in approximately 2% of tumors and is found significantly mutated in endometrial tumors (14%), lung squamous cell carcinoma (5%), lung adenocarcinoma (3%), and head and neck cancer (3%) (16). Mutations in *ARHGAP35* were further confirmed in a study having identified new driver genes in lung carcinogenesis (17). Most *ARHGAP35* mutations are nonsense mutations and frameshift deletions/insertions supporting a tumor-suppressor role for p190A. Given the GAP domain of p190A is localized in the C-terminus, these mutations are expected to produce a nonfunctional protein without GAP activity, as we found for the R997∗ nonsense mutation (6). Besides these mutations, many missense mutations have also been reported throughout the entire *ARHGAP35* sequence. Despite some potentially having no effect on protein function, we have shown that some mutations localized in the PLS and pG2 domain have a strong effect on GAP activity and cell behavior (6). Indeed, the S866F point mutation and the in-frame 865–870 deletion, when ectopically expressed in tumor cells, can induce an increase in GAP activity and alter directed motility of tumor cells (6). It is clear that the effects of such mutations require further investigation. Altogether, combining the identification and characterization of the PLS with the effects of the aforementioned missense mutations leads us to question if p190A could harbor an autoinhibitory folding (18).

Building on our previous results, we aimed to better characterize the involvement of the PLS domain in the regulation of p190A, that is, localization and function. Herein, we confirmed that p190A localizes to various different invadosome organizations, such as dots, rosettes, and linear invadosomes. We found that deletion of the PLS in p190A alters these localizations. Using a global approach and biochemical analyses, we demonstrated that the PLS interacts with a region in close proximity to the GAP domain. Our data thus are consistent with a specific folding of the molecule, involving the PLS and masking the GAP domain. We demonstrated that this interaction is lost upon introduction of S866F and Δ865–870 mutations, providing a molecular explanation to the gain of GAP function observed in cells expressing these mutants. Overall, our findings unveil a new mechanism involved in p190A regulation that identifies the PLS as an inhibitory domain responsible for masking the p190A functional domain.

## Results

### The PLS is required for modulating p190A invadosome localization

**Figure 1:**
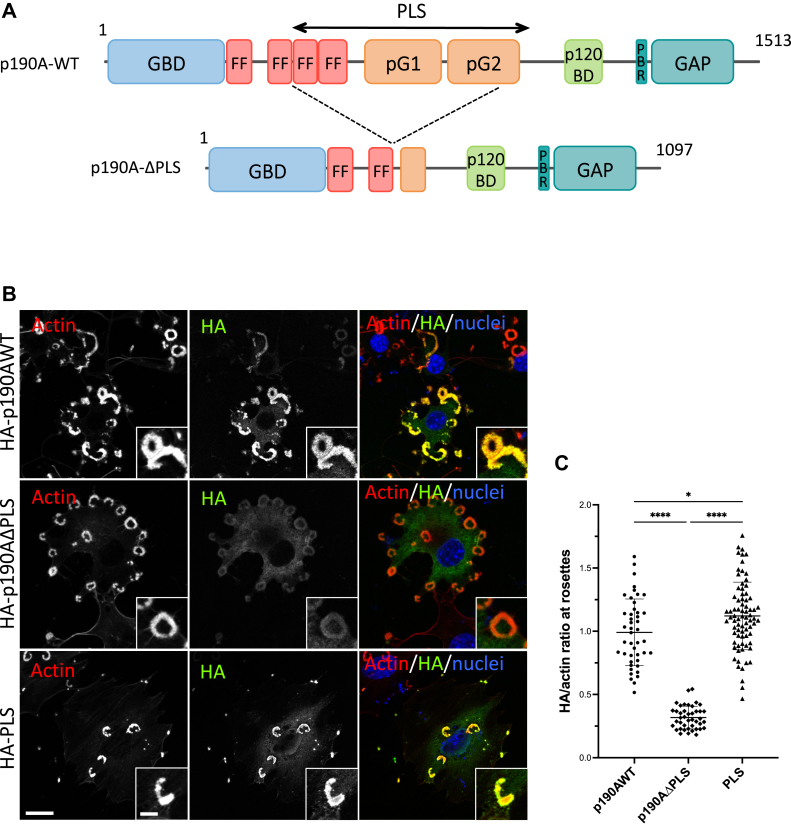

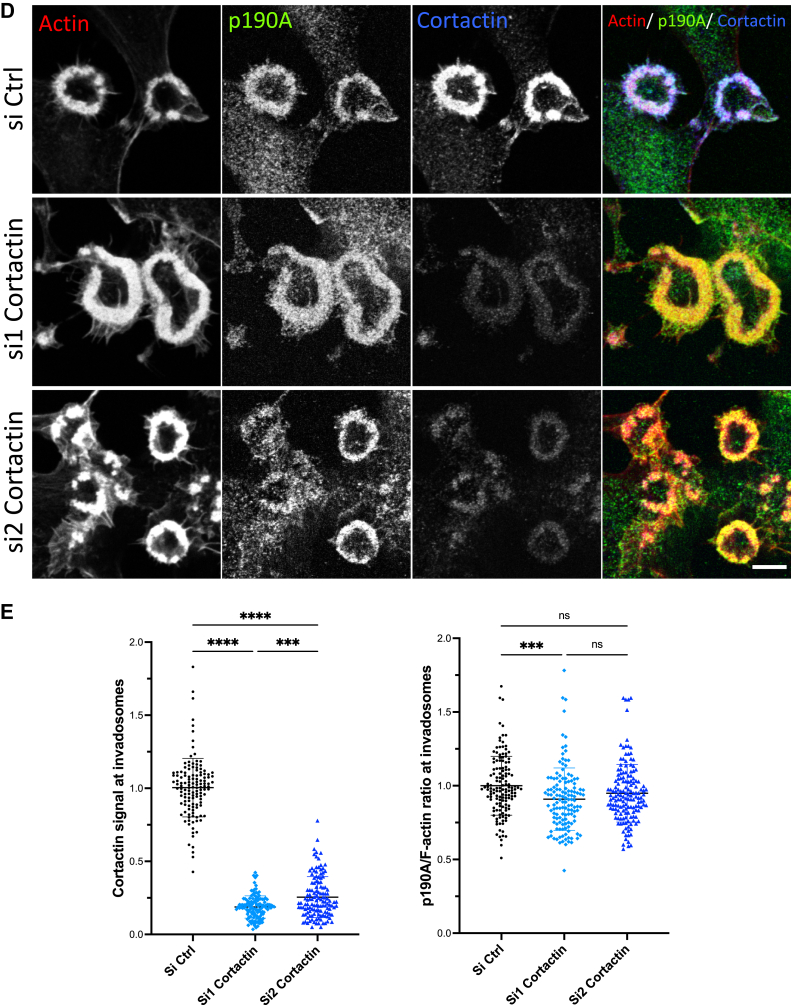
**The PLS is required for modulating p190A invadosome localization.***A*, schematic representation of WT and PLS-truncated p190A. Functional domains are indicated. *B*, NIH-3T3-Src cells were transfected with indicated HA-tagged constructs, plated on glass coverslips, fixed, and stained for F-actin (*red*), HA tag (*green*), and nuclei (*blue*). Bar represents 10 μm. *Boxed regions* are shown as enlarged views; bar represents 3 μm. *C*, graph represents the quantification of the ratio of the HA staining (*green*) on the F-actin staining (*red*) at rosettes determined using imageJ software. Values are expressed as the mean ± SD of three independent experiments. ∗∗∗∗*p* < 0.0001 and ∗*p* < 0.05. *D*, NIH-3T3-Src cells were transfected with siRNAs targeting Cortactin (si1/si2 Cortactin) or control siRNAs (siCtrl). Cells were plated on glass coverslips, fixed, and stained for F-actin (*red*), p190A (*green*), and cortactin (*blue*). Bar represents 2.5 μm. *E*, graphs represent the quantification of the cortactin signal (*left-hand*) and the ratio of the p190 staining (*green*) on the F-actin staining (*red*) (*right-hand*) at rosettes determined using imageJ software. Values are expressed as the mean ± SD of three independent experiments. ∗∗∗∗*p* < 0.0001; ∗∗∗*p* < 0.001 and ns, not significant. GAP, GTPase-activating protein; GBD, GTP-binding domain; p120BD, p120RasGAP-binding domain; PBR, polybasic region; pG1, pseudo-GTPase domain 1; pG2, pseudo-GTPase domain 2; PLS, protrusion localization sequence.

The PLS region (Fig. 1*A*), encompassing two FF motifs, pG1, and pG2, was previously described as a regulator of p190A GAP function and required for targeting p190A to the lamellipodia of migrating cells (6). We analyzed the involvement of the PLS domain in the targeting of p190A to invadosomes. We first confirmed that endogenous p190A localized to different invadosome types regardless of invadosome organization and stimuli. Endogenous p190A was found in spreading Huh6 hepatoblastoma cells in both dot-like invadopodia (cells plated on glass) and linear invadosomes (cells plated on glass coated with type I collagen fibers) but also in v-Src–induced rosettes in NIH-3T3 cells (Fig. S1*A*). We next analyzed the localization of several HA-tagged constructs to check whether the PLS domain is required for p190A localization to invadosomes. Similar to the full-length protein, we found that the PLS alone can localize to invadosomes in NIH-3T3 (Fig. 1, *B* and *C*) and Huh6 cells (Fig. S1*B*). On the contrary, the targeting of a p190AΔPLS mutant (p190A lacking the PLS domain) to invadosomes was strongly affected (Figs. 1, *B* and *C* and S1*B*). These new data confirmed the importance of the PLS in regulating the localization of p190A in addition to its GAP function.

We next investigated the role of the actin-binding protein cortactin in p190A targeting to invadosomes because we previously showed that cortactin is required for p190A targeting to lamellipodia (6). Using the siRNA strategy, we depleted cortactin expression (Fig. S1*C*) and analyzed its impact on p190A localization (Fig. 1, *D* and *E*). As previously described (19), cortactin-deficient cells still formed invadosome rosettes, in a similar way to control cells. We found that endogenous p190A still localized to invadosomes, even if cortactin levels were strongly decreased in rosettes (Fig. 1, *D* and *E*). These data revealed that cortactin is not involved in the recruitment of p190A to invadosomes, which is in contrast to its recruitment to extending lamellipodia.

### The PLS interacts with the C-terminal part of p190A

**Figure 2:**
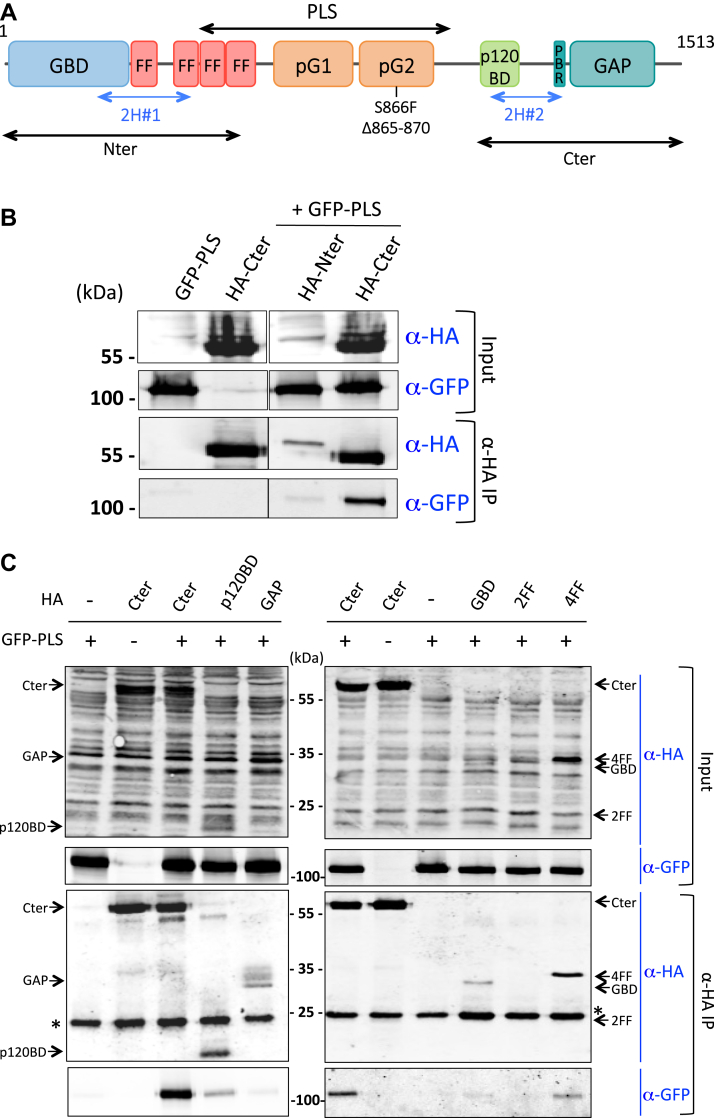

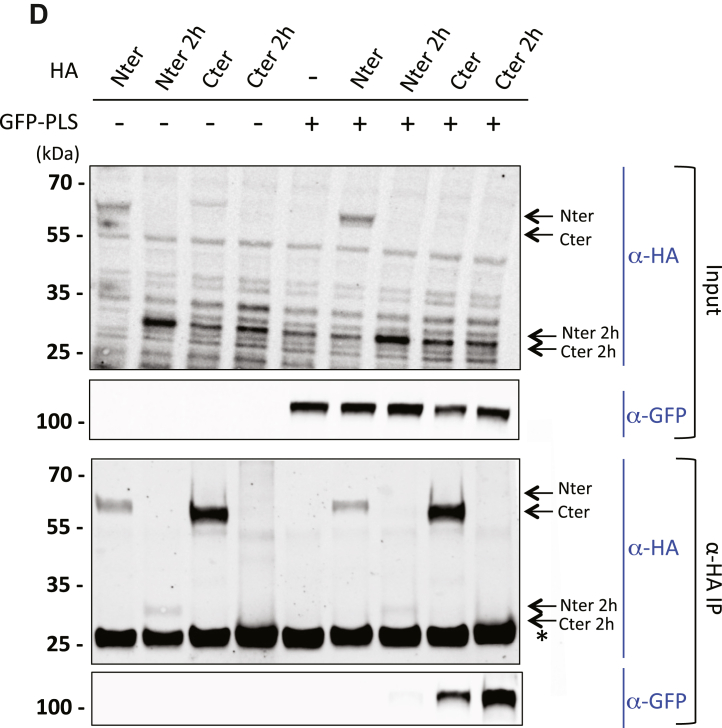
**PLS interacts with the Cter domain of p190A.***A*, schematic representation of p190A showing the PLS, the two regions obtained from the two-hybrid screen, and positions of cancer-associated mutations. *B*–*D*, co-IP of different HA-tagged proteins and GFP-PLS from lysates of HEK293T cells expressing respective tagged proteins. Input and co-IP were analyzed by Western blot with antibodies against HA and GFP tags, as indicated. *Arrows* indicate expected proteins ((*C*) Cter: 60 kDa; p120BD: 15 kDa; GAP: 33 kDa; GBD: 30 kDa; 2FF: 23 kDa; 4FF: 33 kDa, (*D*) Nter: 60 kDa; Nter2h (2H#1 region): 28 kDa; Cter: 55 kDa; Cter2h (2H#2 region): 25 kDa), stars indicate signal of the IgG light chains. Note that HA-Cter2h signal (25 kDa) is fused with the IgG light chain signal resulting in a slightly larger band (*D*, lanes #4 and #9). For each condition, a representative experiment out of three is shown. co-IP, coimmunoprecipitation; Cter, carboxy terminal; GAP, GTPase-activating protein; GBD, GTP-binding domain; Nter, amino terminal; p120BD, p120RasGAP-binding domain; PBR, polybasic region; pG1, pseudo-GTPase domain 1; pG2, pseudo-GTPase domain 2; PLS, protrusion localization sequence.

We performed a yeast two-hybrid screen to identify interacting proteins to understand how this PLS domain could regulate p190A. The PLS domain of p190A was used as a bait in a two-hybrid screen of a highly complex human placenta complementary DNA (cDNA) library (92.3 million interactions). Interestingly, p190A itself was identified as interacting with the PLS. Indeed, 35 clones from human p190A were identified (Table S1) corresponding to two distinct regions of p190A: (i) region 2H#1 (aa 208–418), overlapping the end of the GBD and the first two FF motifs in the N-terminal part of the molecule, and (ii) region 2H#2 (aa 1069–1231), localized in the C-terminal part of p190A (Fig. 2*A*).

We coexpressed GFP-tagged PLS with HA-tagged domains of p190A in HEK293T cells to confirm these results. We used previously generated HA-tagged constructs (6): HA-amino terminal (Nter, aa 1–533) overlapping region 2H#1, and HA-carboxy terminal (Cter, aa 1055–1513) overlapping region 2H#2 (Table S2) (Fig. 2*A*). Using coimmunoprecipitation (co-IP) with anti-HA antibodies, as shown in Figure 2*B*, we observed a strong interaction between the PLS and the Cter domain of p190A. However, despite identifying the region 2H#1 in the two-hybrid screen, interaction between the Nter domain and the PLS was barely detectable (Fig. 2*B*). This weak interaction may be due to the low expression of the HA-Nter construct in HEK293T cells.

To more precisely map the interacting domains of p190A in the N- and C-terminal parts, we performed co-IP experiments between the PLS and smaller p190A domains. Figure 2*C* revealed that while the PLS strongly interacted with the C-terminal domain of p190A, the interaction with the so-called p120-binding domain was reduced and the interaction with the GAP domain was almost absent. Among the domains tested in the N-terminal part of p190A, only a weak interaction between the PLS with the GBD and the four FF domains (4FF) was observed (Fig. 2*C*); no conclusion can be drawn for the two FF domains (2FF) as we were unable to detect 2FF expression. We then generated HA-tagged constructs corresponding to the two regions identified in the two-hybrid screen, that is, regions 2H#1 and 2H#2, and performed co-IPs with GFP-PLS. The 2H#2 region (HA-Cter2h) strongly interacted with the PLS, whereas no interaction with the 2H#1 region was detected (HA-Nter2h) (Fig. 2*D*). This finding indicated that the minimal region of p190A required for interaction with the PLS domain is between aa 1069 and 1231 in the Cter part. Overall, these results show an interaction between two distinct domains of p190A, involving the region close to the GAP domain and the PLS domain, suggesting thus an effect of this interaction on p190A function.

**Figure 3:**
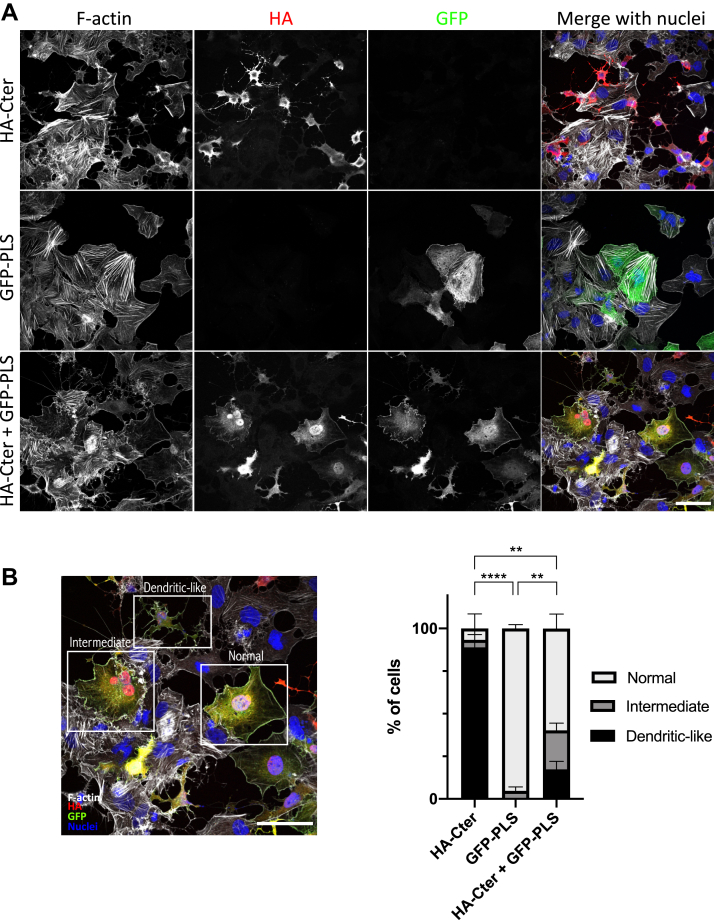
**PLS regulates p190A activity.***A*, Huh7 cells were transfected with HA-tagged Cter and/or GFP-tagged PLS constructs as indicated on the figure. Cells were fixed and stained with phalloidin (*white*), HA (*red*), and GFP (*green*) antibodies, and DAPI (*blue*). Bar represents 15 μm. *B*, quantification of three experiments performed as described in (*A*). For each condition, phenotype is evaluated by using an epifluorescence microscope as normal, intermediate, or dendritic-like, as shown on the reused image from the merge image in the “HA-Cter+GFP-PLS” row of (*A*). Around 50 transfected cells were counted for each of the three experiments. Values are expressed as the mean ± SEM of three independent experiments. Statistical significance was calculated between two conditions with ∗∗*p* < 0.01 and ∗∗∗∗*p* < 0.0001. Cter, carboxy terminal; PLS, protrusion localization sequence.

In order to evaluate the involvement of the PLS in the regulation of p190A GAP activity, we analyzed F-actin organization as a read-out of Rho GTPase activity. Huh7 cells were previously used to identify the PLS domain (6) and are highly sensitive to RhoGAP activity increase. Indeed, we found that overexpression of the HA-tagged Cter domain of p190A, containing the GAP domain, strongly affected F-actin organization. The majority of Huh7 cells exhibited long membrane protrusions with a decrease in spreading, the so-called dendritic-like phenotype (Fig. 3, *A* and *B*). We thus questioned whether coexpression of the PLS with the Cter domain could rescue this phenotype by interacting with the Cter domain. To investigate this, we expressed the GFP-tagged PLS domain in Huh7 cells either alone or in combination with the HA-tagged Cter. Expression of the PLS domain alone did not change Huh7 cell phenotype, whereas coexpression of the PLS domain with the Cter domain strongly reduced the dendritic-like phenotype observed with HA-Cter expression (Fig. 3, *A* and *B*). Some cells also harbored an intermediate phenotype under Cter and PLS domain coexpression, suggesting a partial rescue of the normal phenotype (Fig. 3*B*). Therefore, our data demonstrate that through its interaction with the Cter domain, the PLS is able to inhibit the GAP activity of p190A.

### Phosphorylation is not involved in PLS/Cter interaction

**Figure 4:**
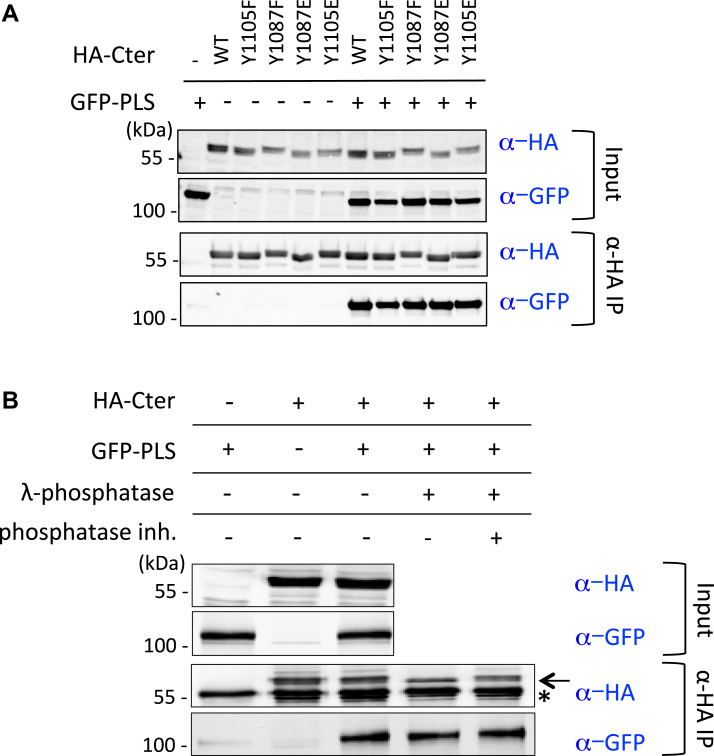

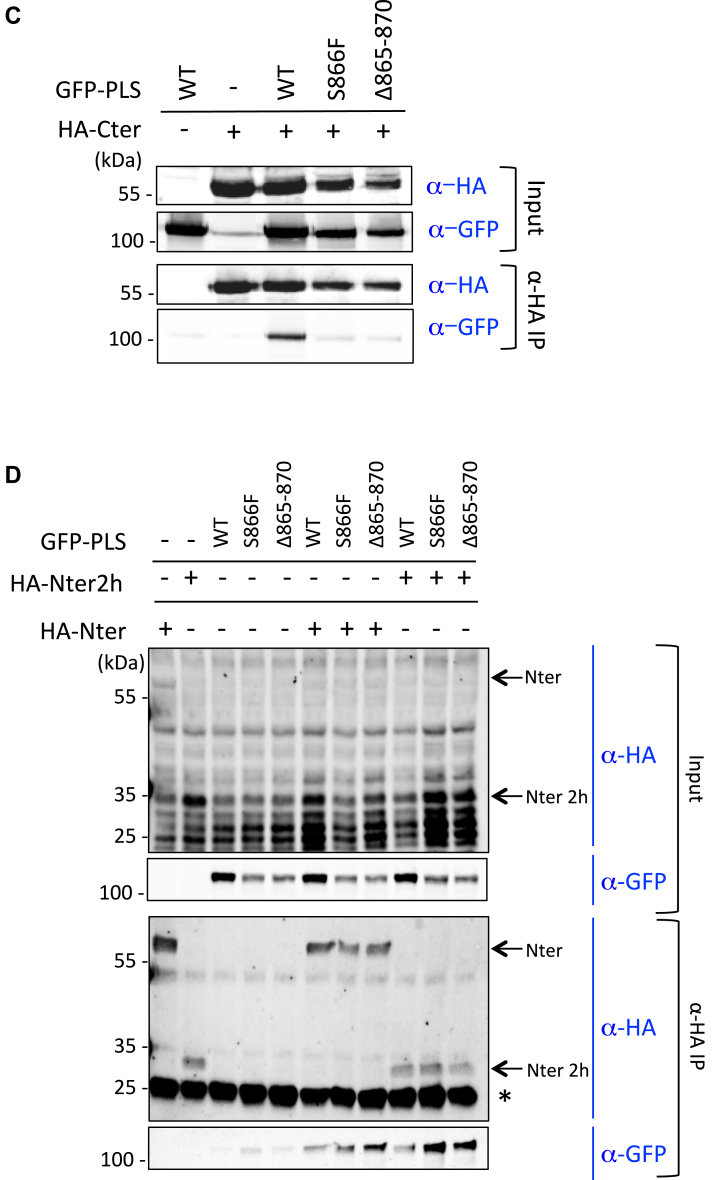
**Cancer-associated mutations in PLS abolish PLS/Cter interaction and increase PLS/Nter interaction.***A*–*D*, HEK293T cells were cotransfected with indicated constructs and co-IP were performed using HA-coupled beads. Cell extracts were analyzed by Western blot before (Input) or after co-IP (α-HA IP) using anti-HA and anti-GFP antibodies. *B*, phosphorylation is not implicated in Cter/PLS interaction. Cells were cotransfected as indicated and treated with phosphatase with or without phosphatase inhibitor before co-IP. *D*, *arrows* indicate expected proteins (Nter: 60 kDa; Nter2h: 28 kDa), *stars* indicate signal of the IgG light chains. *A*–*D*, a representative experiment out of three is shown. co-IP, coimmunoprecipitation; Cter, carboxy terminal; Nter, amino terminal; PLS, protrusion localization sequence.

Some amino acids localized in the C-terminal part and notably in the 2H#2 region of p190A have already been recognized as involved in the function and regulation of the protein. In fact, Y1087 and Y1105 were found implicated in p120RasGAP binding (13, 14). We attempted to check whether these amino acids could regulate or modulate the aforementioned interactions we demonstrated using phosphosite mutants. We introduced Y1105F, Y1087F, Y1087E, or Y1105E mutations by site-directed mutagenesis into the HA-Cter construct. We then performed co-IP experiments using these mutants and GFP-PLS (Fig. 4*A*). No change in the PLS/Cter interaction was observed using either the WT or mutated Cter domains. This result suggests that phosphorylation of these two specific residues is not implicated in the binding of the Cter domain with the PLS. We next considered whether phosphorylation was generally necessary for this interaction. Figure 4*B* shows that phosphatase treatment did not modify the interaction between the Cter and PLS domains, implying that phosphorylation is not involved in this molecular interaction.

### Cancer-associated mutations alter PLS/Cter interaction

The S866F point mutation and the small deletion of five amino acids (Δ865–870; thus including S866) were both previously described as cancer-associated mutations and as mutations mimicking PLS deletion in p190A function (6). Given both mutations are localized within the PLS domain of p190A (Fig. 2*A*), we analyzed their effect on the PLS/Cter interaction. To do so, we introduced each mutation in the GFP-tagged PLS construct and monitored interaction with HA-tagged Cter by co-IP. Interestingly, we showed that introducing the S866F mutation or deleting the five amino acids (Δ865–870) in the PLS abolished the interaction of the PLS domain with the Cter region of p190A (Figs. 4*C* and S2). We further examined the effect of these mutations on the weak PLS/Nter interaction. Conversely, we found that both mutations increased the interaction between the Nter and the PLS domain (Fig. 4*D*). The same results were obtained when co-IP experiments were performed with the Nter2h corresponding to the 2H#1 region (Fig. 4*D*). These findings suggest that both mutations could alter the conformation of p190A by modifying domain interactions. This would also imply that these mutations lead to the release of Cter and the PLS domain interaction, in turn unmasking the GAP domain and simultaneously increasing the interaction between the Nter part of p190A and the PLS.

## Discussion

Herein, we confirmed that the PLS of p190A is an important functional domain of p190A. This PLS domain, encompassing two FF motifs, pG1, and pG2, was previously identified in a structure/function analysis as the PLS of p190A (6). Indeed, the PLS is both required and sufficient for p190A targeting to the lamellipodia and leading edges of migrating cells. Given invadosomes are also actin-based protrusions, where p190A has been shown to regulate Rho GTPases (such as RhoC/TC10 activities) (20, 21), we analyzed the involvement of the PLS in targeting p190A to invadosomes. We first complemented available data on invadosome localization of p190A (8, 9, 22, 23). We demonstrated that WT p190A can be found in all invadosome structure types, such as rosettes in vSrc-transformed fibroblasts as well as in dots and linear invadosomes in Huh6 tumor cells. Using a truncated version of p190A, we demonstrated that the PLS is also required and sufficient for p190A targeting to these invadosome structures. However, the molecular mechanism of targeting remains to be explored as, unlike lamellipodia (6), cortactin is not required to target p190A to these invasive actin-based structures.

**Figure 5:**
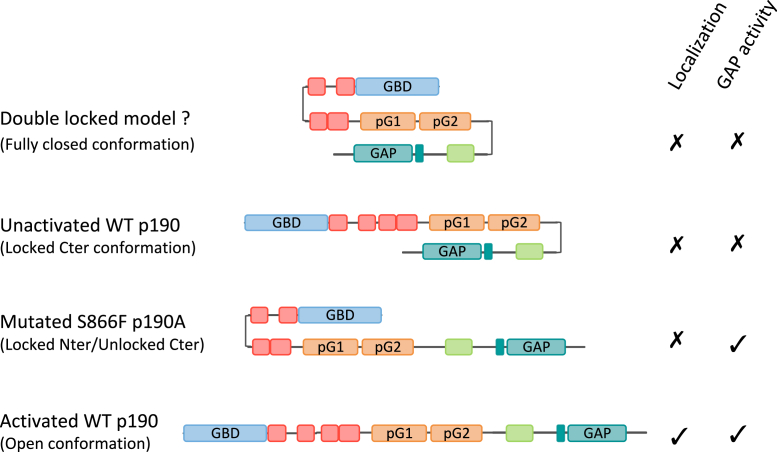
**Model of p190A autoinhibition.** Representation of p190A protein exhibiting an intramolecular interaction involving the PLS domain. In the inactivated WT protein, PLS is interacting either with the Nter and the Cter or the Cter domain alone in a locked Cter conformation masking the GAP domain. In the mutated protein, the Cter is not interacting anymore with the PLS leading to unmasking the GAP domain and increasing the GAP activity. The Nter domain would interact with the PLS preventing the correct localization of the protein. A fully activated protein may require an open conformation to allow proper localization and GAP activity. Cter, carboxy terminal; GAP, GTPase-activating protein; Nter, amino terminal; PLS, protrusion localization sequence.

We previously reported that the PLS is required for the negative regulation of p190A RhoGAP activity. We indeed described that RhoA activity decrease is stronger in cells expressing p190AΔPLS *versus* p190AWT. This strong p190AΔPLS effect arises from the drastic increase in the affinity of p190A for active RhoA (6). Thus, these data strongly support a PLS-mediated inhibition of the GAP domain. Due to the presence of two pseudo-GTPase domains (pG1 and pG2) in the PLS, we previously hypothesized that an intramolecular interaction could occur between pG1 or pG2 and the GAP domain (defined as a GTPase-interacting domain) (4, 18). We performed a global approach to identify PLS-interacting proteins. The two-hybrid screen we performed was rather stringent as we only recovered 19 proteins in the very high to medium-high confidence ranges. Despite cortactin being previously identified as a protein interacting with the PLS (6), cortactin was not recovered in this screen. However, we identified two p190A regions that do interact with the PLS. Using co-IP, we showed that the region corresponding to aa 1069 to 1231 strongly interacts with the PLS. Interestingly, this region excludes the GAP domain but overlaps the p120RasGAP-binding domain of p190A (aa 1055–1143) (13) and the polybasic region identified as crucial for p190A phospholipid binding (24, 25). Experiments performed in Huh7 cells strongly suggested that the PLS could inhibit the GAP function of p190A. Thus, our results are consistent with the determination of the PLS as an inhibitory domain responsible for p190A GAP activity repression. Given deletion of PLS increased the affinity of p190A for active RhoA, the PLS domain may act in *cis* through an intramolecular folding that masks the p190A catalytic domain (Fig. 5); this locked Cter conformation would result in an inactivated form of p190A. An autoinhibitory control of GAP activity due to protein folding has already been described for several other GAPs, including p50RhoGAP, oligophrenin, Abr, and chimerins (26). Furthermore, the Bin-Amphiphysin-Rvs domain of oligophrenin acts as a *cis*-acting inhibitory element that masks the GAP domain to prevent the downregulation of Rho GTPases (27). It is noteworthy that we cannot exclude among our findings here a role of the PLS in a *trans*-inhibition *via* an intermolecular interaction, even if to date, p190A has never been found acting as a dimer.

The hypothesis of this autoinhibition mechanism raises questions regarding the release of the autoinhibited state. Indeed, signals that may act as molecular conformation switches must activate p190A at its correct localization. Nevertheless, such signals remain to be identified. Protein partners and posttranslational modifications, such as phosphorylation, are initial evident candidates. In this line, previous studies have proposed the involvement of Rac1 and Rnd3 for enhancing the RhoGAP activity of p190B, a paralog of p190A (28). However, neither Rac1 nor Rnd3 were able to alter the p190A PLS/Cter interaction; the same result was also obtained using p120RasGAP (data not shown). We additionally exclude the involvement of phosphorylation in this study. The PLS/Cter interaction of p190A was not modified by neither mutations of tyrosine phosphosites in the p120RasGAP-binding domain nor phosphatase treatment.

Our data highlight serine 866 as an important residue for p190A regulation. Indeed, the cancer-associated S866F missense mutation and the Δ865–870 deletion abolished the PLS/Cter interaction as shown by co-IP. Both mutations were previously shown to mimic the deletion of the PLS domain with a mislocalization and an increase in GAP activity. Thus, these new data provide a molecular explanation for the observed phenotypes and define these mutant proteins as constitutively active forms of p190A with an unmasked Cter domain (Fig. 5). Since phosphorylation is frequently involved in functional regulation, it was tempting to hypothesize that phosphorylation of serine 866 maintains p190A in its closed conformation, with dephosphorylation activating p190A. However, consistent with our phosphatase treatment data here, mass spectrometry analysis revealed that S866 is not phosphorylated in WT p190A (data not shown). The closed conformation of p190A, involving the PLS/Cter interaction, is likely not the only mechanism regulating p190A folding. Indeed, our two-hydrid screen results demonstrated that the Nter part of p190A may under some circumstances interact with the PLS. We found that the missense S866F mutation and Δ865–870 deletion strongly altered p190A conformation, favoring the PLS/Nter interaction instead of the PLS/Cter interaction, with a locked Nter and unlocked Cter conformation (Fig. 5).

Altogether, our new data support an intramolecular model (Fig. 5) in which p190A is found in a closed conformation. This conformation may be opened upon stimulation, leading to the translocation and full activation of p190A restricted to actin-based structures, such as lamellipodia and invadosomes. Thus, the PLS domain is at the basis of a dual-effect system with independent membrane-localized and GAP-inhibitory functions that may operate simultaneously. In addition to S866F, other missense cancer-associated mutations may alter this mechanism and result in altered GAP function equivalent to nonsense mutations.

## Experimental procedures

### Cell lines

HEK293T, NIH-3T3-vSrc, and Huh7 cell lines were cultured in Dulbecco’s Modified Eagle’s Media containing 4.5 g/l glucose and Glutamax (Gibco) supplemented with 10% fetal calf serum (Eurobio). Hepatoblastoma Huh6 cells were maintained in Dulbecco’s Modified Eagle’s Media containing 1 g/l glucose and Glutamax (Gibco) supplemented with 10% fetal calf serum (Eurobio). All cells were maintained at 37 °C in a 5% CO_2_ humidified atmosphere. HEK293T and Huh7 cell lines were purchased from American Type Culture Collection. Huh6 cells were a gift from C. Perret (Cochin Institute) and NIH-3T3-vSrc cells from S. Courtneidge (Burham Institute for Medical Research). Cell lines were confirmed for the absence of *mycoplasma* by PCR and authenticated by STR Matching analysis (American Type Culture Collection).

### Antibodies and reagents

Mouse monoclonal (clone 12CA5) and rat monoclonal (clone 3F10) anti-HA antibodies were purchased from Roche. Several anti-HA antibodies were screened, all showing a strong background by Western blot. Mouse anti-p190A (clone D2D6) and rabbit polyclonal anti-GFP antibodies were obtained from Sigma, mouse anti-p190A antibody (clone 30) from BD. Rabbit polyclonal and mouse monoclonal (clone 4F11) anti-cortactin antibodies were purchased from Cell Signaling and Millipore, respectively. Rabbit (FL-335) and mouse (D-6) anti-GAPDH antibodies were obtained from Santa Cruz Biotechnology. Antimouse IRDYE680 and anti-rabbit IRDYE800 secondary antibodies were obtained from Eurobio Scientific. FluoProbes 488-phalloidin and 488-labeled secondary antibodies and 547-labeled secondary antibodies were purchased from Interchim. Hœscht 405 (34580, Invitrogen) was used to stain nuclei.

### Two-hybrid screen

A bait cDNA construct (pB27 bait vector) expressing the PLS (aa 380–971) from *Rattus norvegicus ARHGAP35* fused to the lexA DNA-binding domain was used to screen for prey clones in a human placenta cDNA library (pP6 prey vector). This screen was performed by Hybrigenics services (Evry) using the ULTImate Y2HTM screen.

### Transfection

DNA transfections in HEK293T cell line were performed using Lipofectamine 2000 (Thermo Fisher Scientific), Huh6 and Huh7 cells were transfected using PromoFectin-hepatocyte transfection reagent (Promocell), and NIH-3T3-vSrc cells with JetPrime (PolyPlus Transfection) according to the manufacturer’s instructions. siRNA oligos were purchased from Eurofins Genomics and transfected into NIH-3T3-vSrc cells with JetPrime (PolyPlus Transfection) according to the manufacturer’s protocol. Si1 Cortactin targets cortactin mRNA at 5′-GGAACACAUCAACAUUCACTT-3′ and Si2 Cortactin targets at 5′-AAGCUUCGAGAGAAUGUCUUC-3′. Control siRNA corresponds to AllStars Negative control from Qiagen.

### Plasmid constructs

The pKH3-p190A-WT containing the rat full-length p190RhoGAP (GenBank under accession no. M94721) was a generous gift from I. Macara (Vanderbilt University). The two truncated p190A constructs (Nter2h and Cter2h) were engineered by PCR using the rat full-length p190RhoGAP as matrix and subcloned into pKH3 expression vector using BamHI and EcoRI restriction sites. Point mutations were generated in the parental vector pKH3-p190A using site-directed mutagenesis with the QuikChange II XL kit according to the manufacturer’s protocol (Agilent Technologies). The two mutated PLS constructs (PLS S866F and PLS Δ865–870) were generated by site-directed mutagenesis in the rat GFP-PLS construct. The four mutated Cter constructs (Cter Y1087F, Cter Y1087E, Cter Y1105F, and Cter Y1105E) were generated by site-directed mutagenesis in the rat HA-Cter construct. All constructs were verified by DNA sequencing. All other p190 constructs (truncated and/or mutated p190A constructs) were previously generated and published (6). All constructs used in this study are summarized in Table S2. All primers used for these constructs are listed in Table S3.

### Western blot analysis and immunoprecipitation

Twenty four hours after transfection, cells were scraped off on ice and homogenized in Tris–HCl lysis buffer (50 mM Tris–HCl, pH 7.4, 150 mM NaCl, and 0.1% NP-40) with protease and phosphatase inhibitors. Cell lysates were centrifuged at 10,000*g* for 10 min to erase cellular debris and nuclei. Lysates were denatured with Laemmli loading buffer containing 2.5% 2-β-mercaptoethanol for 5 min and analyzed by SDS-PAGE by blotting them onto nitrocellulose membranes. Blots were incubated overnight at 4 °C or for 1 h at room temperature with primary antibodies and then incubated with infrared fluorescent dye-conjugated secondary antibodies (Eurobio). Activity was visualized with the Odyssey infrared imaging system (LI-COR Biosciences) and the Chemidoc infrared imaging system (Bio-Rad) and analyzed using ImageJ image analysis software. Anti-HA affinity matrix was obtained from Roche for immunoprecipitation assays and used following the manufacturer’s protocol. Briefly, cells were extracted in the Tris–HCl buffer (50 mM Tris–HCl, pH 7.4, 150 mM NaCl, and 0.1% NP-40) supplemented with protease and phosphatase inhibitors and centrifuged for 10 min at 10,000*g*. One milligram of the supernatant was incubated for 30 min with the beads. Bead pellets were washed three times with Tris–HCl buffer, resuspended in loading buffer before analysis by SDS-PAGE.

### λ-Phosphatase treatment

Protein lysates were incubated with λ-phosphatase (New England Biolabs) with or without the recommended amount of the Halt Phosphatase Inhibitor Mixture (Thermo Fisher Scientific) for 30 min at 30 °C. Samples were washed six times with buffer, according to the manufacturer’s protocol, prior to co-IP and subsequent SDS-PAGE analysis.

### Collagen I coating

To induce linear invadosome formation, coverslips were prepared as previously described (29). Coverslips were coated with 0.5 mg/ml type I collagen (BD Biosciences) in Dulbecco’s phosphate buffered saline (Gibco). Collagen-coated coverslips were incubated for 4 h at 37 °C, after which they were washed gently in PBS (Invitrogen). Cells were seeded and fixed after 5 h of incubation at 37 °C before staining.

### Immunofluorescence and confocal imaging

Twenty four hours after transfection on glass coverslips, cells were fixed with 4% paraformaldehyde for 10 min at RT and permeabilized with 0.2% Triton X-100 for 10 min before incubation with various antibodies, as previously described (30). After staining, coverslips were mounted on slides with Fluoromount G mounting medium. Cells were imaged using an SP5 confocal microscope (Leica Biosystems) using a 63×/NA 1.4 Plan Neofluor objective lens and the LAS-AF-Lite 2.4.1 acquisition software (Leica Biosystems). To prevent contamination among fluorochromes, each channel was imaged sequentially using the multitrack recording module before merging. Images were processed using LAS-AF-Lite 2.4.1 (Leica Biosystems) or ImageJ software (National Institutes of Health). For the quantification of the invadosome images, signal intensities were measured using imageJ software on images captured with the same laser intensities. Signals of ≥45 invadosome rosettes were quantified for each condition in three independent experiments. For the quantification of the dendritic-like phenotype, around 50 transfected cells in each condition were analyzed through actin staining and their morphology using a Zeiss Axioplan 2 epifluorescence microscope. Scoring was performed, under the microscope, in three independent experiments, by two independent persons, in a blind manner.

### Statistical analysis

Statistical analysis was performed with Prism software (GraphPad Software) and data are presented as mean ± SEM of at least three independent experiments. Comparisons between two groups were analyzed by *t* test. Significance was accepted for values where ∗*p* < 0.05; ∗∗*p* < 0.01; ∗∗∗*p* < 0.001; and ∗∗∗∗*p* < 0.0001.

## Data availability

All data are contained in the article.

## Supporting information

This article contains supporting information.

## Conflicts of interest

The authors declare that they have no conflicts of interest with the contents of this article.

## Supporting information

Supplemental Figures and Tables Legends

Supplemental Figure S1A

Supplemental Figure S1B and C
