# Supplementary material for: Identification of an inhibitory domain in GTPase-activating protein p190RhoGAP responsible for masking its functional GAP domain
Source: J Biol Chem. 2022 Dec 11;299(1):102792. doi: 10.1016/j.jbc.2022.102792 (PMC9840978; doi:10.1016/j.jbc.2022.102792)
Supplement: Supplemental Figures and Tables Legends [file mmc2.docx]

**SUPPORTING INFORMATION**

**SUPPLEMENTARY TABLES**

**Supplementary Table I**: ARHGAP35/GRLF1 clones obtained in the 2Hydrid screen

**Supplementary Table II**: *ARHGAP35* constructs used in this study. Numbering corresponds to the rat *ARHGAP35* cDNA (GenBank under accession no. M94721) that was used.

**Supplementary Table III**: *ARHGAP35* primers used in this study.

**SUPPLEMENTARY FIGURE LEGENDS**

# **Supplementary Figure 1: PLS is necessary for p190A invadosome localization**

(A) NIH-3T3-Src cells were plated on glass coverslip (upper panel) and Huh6 cells were plated on coverslips treated (bottom panel) or not (middle panel) with type I collagen. All cells are stained for endogenous F-actin (red), p190A (green), and nuclei (blue). Merge images are represented. (B) Huh6 cells transfected with indicated HA-tagged constructs were plated on type I collagen-coated coverslips to induced linear invadosomes. Cells were then fixed and stained for F-actin (red), HA tag (green), and nuclei (blue). Bar: 10 μm. (A-B) Boxed regions are shown as enlarged views. Bar: 3 μm. (C) NIH-3T3-Src cells were transfected with siRNAs targeting Cortactin (si1/si2 Cortactin) or control siRNAs (siCtrl). Cell extracts were analyzed by Western-blot using anti-Cortactin and anti-GAPDH antibodies. GAPDH was used as the loading control. The graph shows the quantification of Cortactin expression assayed by western blot (n = 3). Shown is the relative expression when compared to the loading control (GAPDH). Error bars: SD of three independent experiments. Significance was determined with One-way ANOVA test; *****P* < 0.0001; ns, non significant.

# **Supplementary Figure 2: Cancer-associated mutations in PLS abolish PLS/Cter interaction.**

Quantification of the interaction between PLS and Cter domains. Quantification was performed on 3 experiments as follows: band intensity of the GFP staining (IP condition normalized to the input condition) divided by band intensity of the HA staining (IP condition normalized to the input condition). Mutated values were reported to WT values. Statistical significance was calculated between mutant and WT conditions using *t*-test with ***, *P* < 0.001; **, *P* < 0.01.
