# Supplementary figures and images for: Identification of an inhibitory domain in GTPase-activating protein p190RhoGAP responsible for masking its functional GAP domain
Source: J Biol Chem. 2022 Dec 11;299(1):102792. doi: 10.1016/j.jbc.2022.102792 (PMC9840978; doi:10.1016/j.jbc.2022.102792)

# Supplementary Figure 1

**A**

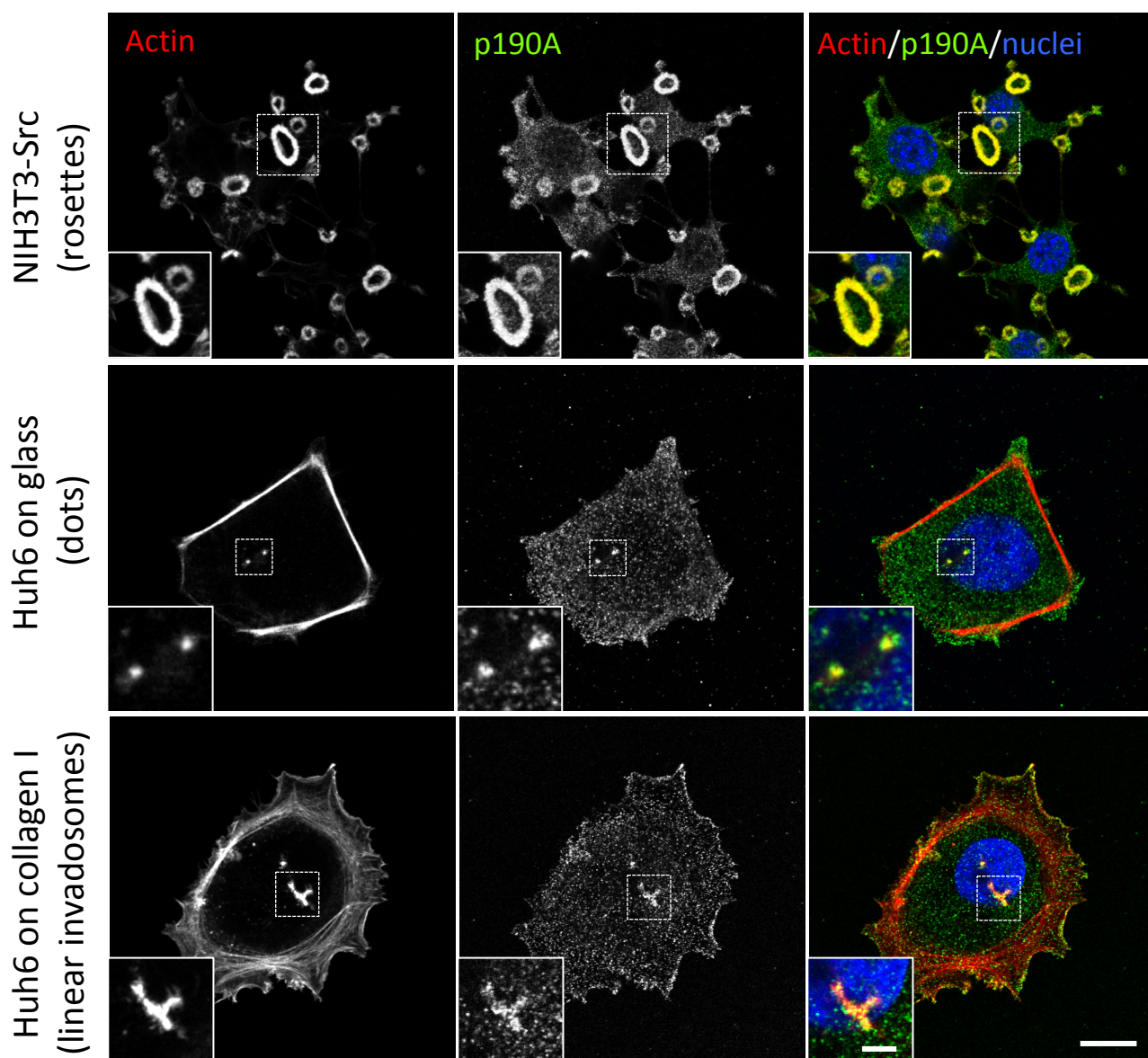

Supplement: Supplemental Figure S1A [file mmc3.pdf]

# Supplementary Figure 1

**B**

Huh6 cells on type I collagen

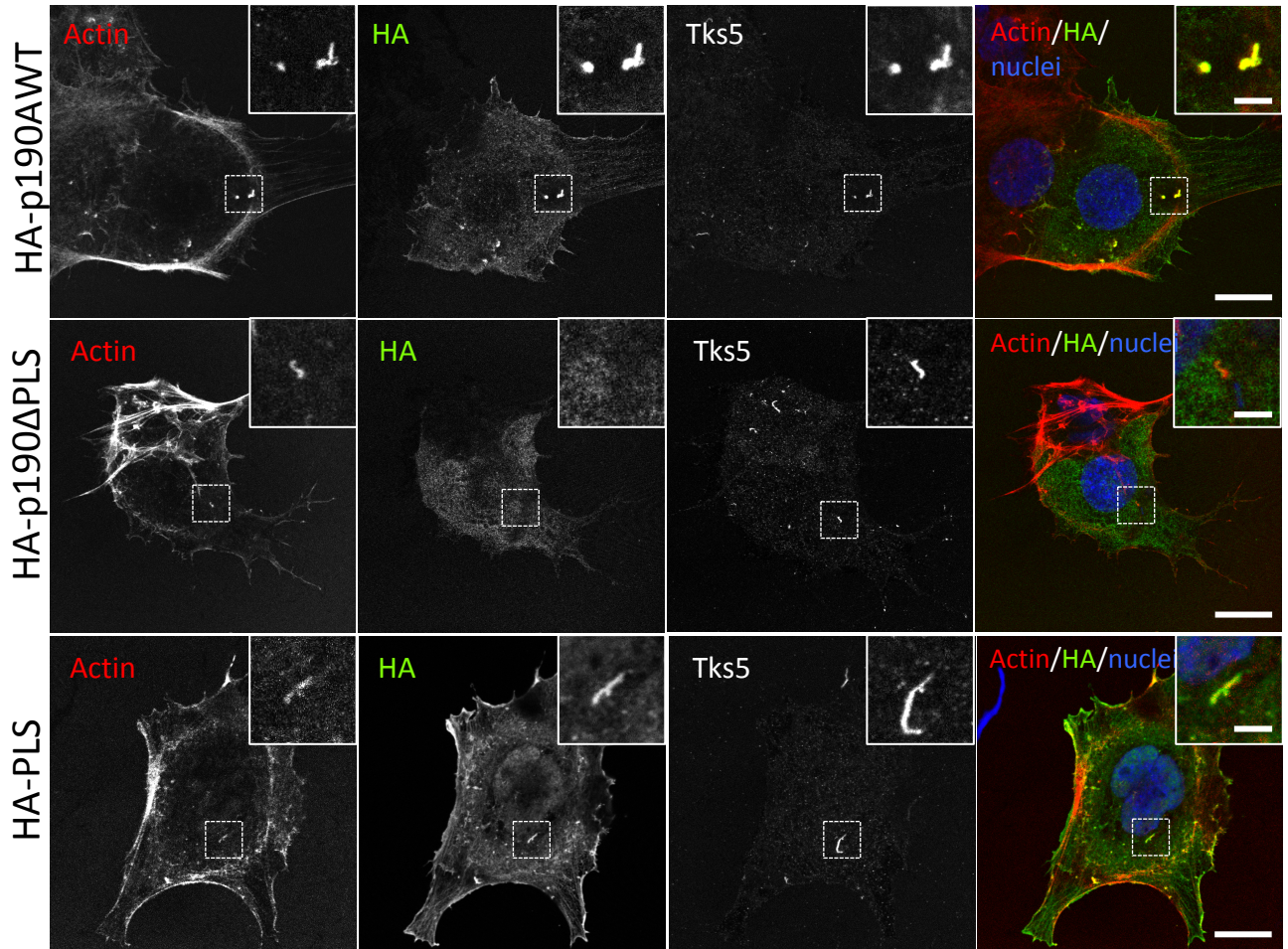

**C**

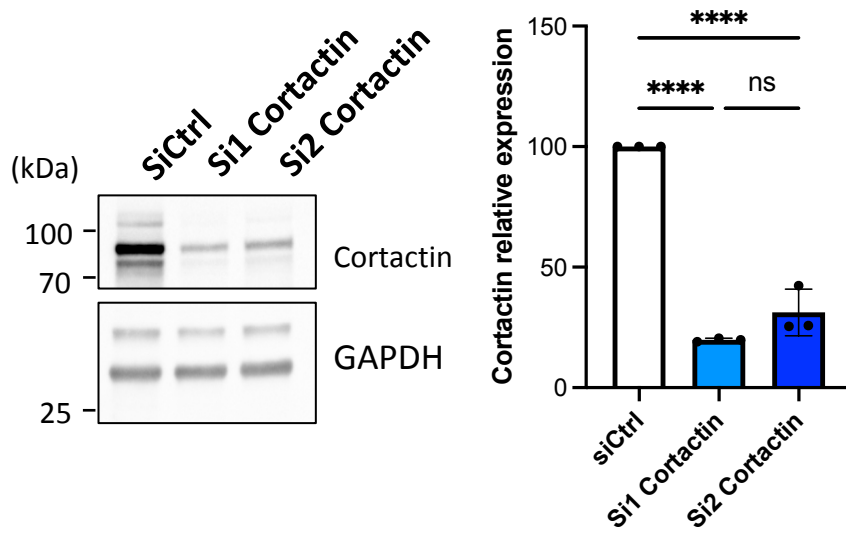

Supplement: Supplemental Figure S1B and C [file mmc4.pdf]

*Supplementary Figure 2*

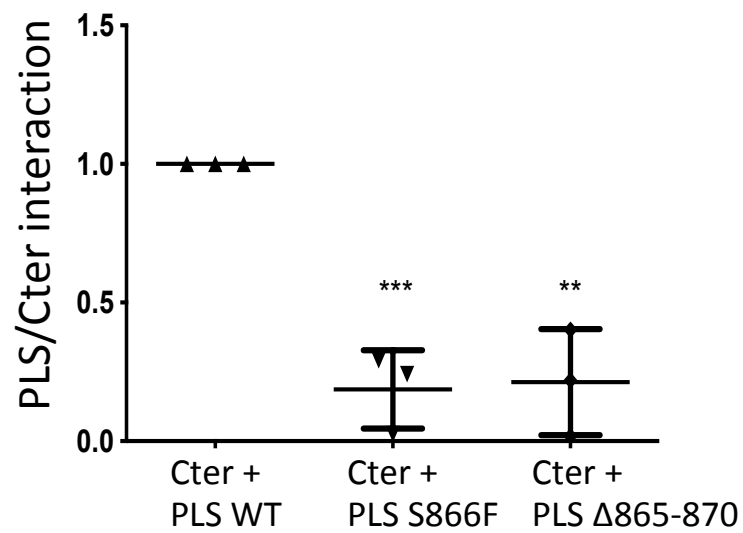

Supplement: Supplemental Figure S2 [file mmc5.pdf]
